# *Arabidopsis AtMSRB5* functions as a salt-stress protector for both Arabidopsis and rice

**DOI:** 10.3389/fpls.2023.1072173

**Published:** 2023-03-22

**Authors:** Yu-Si Cai, Jung-Long Cai, Jent-Turn Lee, Yi-Min Li, Freta Kirana Balladona, Dewi Sukma, Ming-Tsair Chan

**Affiliations:** ^1^ Graduate Program of Translational Agricultural Sciences, National Cheng Kung University and Academia Sinica, Tainan, Taiwan; ^2^ Academia Sinica Biotechnology Center in Southern Taiwan, Agricultural Biotechnology Research Center, Academia Sinica, Tainan, Taiwan; ^3^ Department of Agronomy & Horticulture, Faculty of Agriculture, IPB University, Bogor, Indonesia

**Keywords:** Arabidopsis, methionine sulfoxide reductase, Na +/K + homeostasis, salt tolerance, transgenic rice

## Abstract

Salinity, drought and low temperature are major environmental factors that adversely affect crop productivity worldwide. In this study we adopted an activation tagging approach to identify salt tolerant mutants of Arabidopsis. Thousands of tagged Arabidopsis lines were screened to obtain several potential mutant lines resistant to 150 mM NaCl. Transcript analysis of a salt-stress tolerance 1 (sst1) mutant line indicated activation of AtMSRB5 and AtMSRB6 which encode methionine sulfoxide reductases. Overexpression of AtMSRB5 in Arabidopsis (B5OX) showed a similar salt tolerant phenotype. Furthermore, biochemical analysis indicated stability of the membrane protein, H+-ATPase 2 (AHA2) through regulation of Na+/K+ homeostasis which may be involved in a stress tolerance mechanism. Similarly, overexpression of AtMSRB5 in transgenic rice demonstrated a salt tolerant phenotype *via* the modulation of Na+/K+ homeostasis without a yield drag under salt and oxidative stress conditions.

## Introduction

In nature, plants constantly encounter a wide range of biotic and abiotic stresses which adversely affect crop productivity with losses accumulating to economically damaging levels. Abiotic stress in the broadest sense encompasses cold, drought and salt stress. Crops have evolved complex physiological and biochemical sensing and responsive systems to cope with various physical environments. The products of these genes may participate in the generation of regulatory molecules such as plant hormones, abscisic acid (ABA), ethylene and salicylic acid (SA) (Lee et al., 2003). These regulatory molecules modulate secondary messengers such as Ca^2+^ initiating the protein phosphorylation cascade that finally targets proteins directly involved in cellular protection or the transcriptional factors controlling specific sets of stress-regulated genes (Xiong et al., 2002).

Soil salinity adversely affects crop productivity and quality. High concentration of NaCl impairs electron transport in photosynthesis and causes increased formation of reactive oxygen species (ROS) (Mishra et al., 2013). Previous studies in four rice varieties differing in salt-tolerance, showed the involvement of a ROS mechanism (Dionisio-Sese and Tobita, 1998). Similarly, Sakamoto et al. (2008) reported that a salt tolerant phenotype in *Arabidopsis* expressing an ankyrin-repeat protein encoded by *Arabidopsis increased tolerance to NaCl 1 (ITN1)*, may be due to ROS regulation. It has been demonstrated that plants overexpressing *salt overly sensitive 1* (*SOS1*) overcome salt stress and the salt-induced stability of *SOS1* mRNA is mediated by ROS (Shi et al., 2003; Chung et al., 2008). A haem oxygenase (HO), an important component of the antioxidant system, modifies salinity tolerance by regulating the Na^+^/K^+^ ratio *via* modulating SOS1 and H^+^-ATPases (AHAs) (Bose et al., 2013). Overexpressing tonoplast intrinsic proteins (TIPs) has been shown to improve drought and salt tolerance, suggesting that TIPs revealed differential regulation in response to environmental constraints (Rodrigues et al., 2016). TIPs are known to be targeted to the vacuolar membrane and facilitate water and hydrogen peroxide (H_2_O_2_) across this subcellular compartment (Maeshima, 2001). Therefore, detoxification of ROS has been considered to be an important part of engineering salt tolerance in plants (Chinnusamy et al., 2005; Moller et al., 2007; Das, 2013).

Although ROS play a significant role in relaying signaling molecules under appropriate stress conditions, their presence at high concentration is also detrimental to cells (Apel and Hirt, 2004), causing oxidative damage to membrane lipids, nucleic acids, and proteins. The methionine (Met) residues of proteins are particularly susceptible to ROS, whose presence results in the formation of methionine sulfoxide (MetO) (Davies, 2005). MetO can alter the folding conformation of the native protein and affects its solubility, stability and biological functions (Friguet, 2006). Fortunately, the oxidation of methionine can be readily reversed by the methionine sulfoxide reductase (MSR) system. Depending on the type of enantiomers, the MetO present in its S- and R-form are reduced by the enzymes MSRA and MSRB, respectively (Boschi-Muller et al., 2005; Hansel et al., 2005).

The methionine sulfoxide reductases (MSR) have been reported to have roles in various stress tolerance (Romero et al., 2004; Kwon et al., 2007; Oh et al., 2010; Li et al., 2012; Tarrago et al., 2012; Laugier et al., 2013; Lee et al., 2014; Lee et al., 2015; Roy and Nandi, 2016). Analysis of rice and *Arabidopsis* genomes, revealed the presence of three *MSRB* genes in rice (*OsMSRB1*, *OsMSRB3*, and *OsMSRB5*) and nine in *Arabidopsis* (*AtMSRB1-9*) (Rouhier et al., 2006; Xiao et al., 2018). Kwon et al. (2007) reported that the constitutive expression of *Arabidopsis MSRB3* enhanced plant tolerance to oxidative stress and freezing temperatures during cold acclimation. The *msrb1/msrb2* double mutant shows retarded growth and development under high-light and low-temperature conditions (Laugier et al., 2010). The constitutive expression of *Arabidopsis MSRA4* enhanced plant tolerance to methyl viologen (MV)- and ozone-induced oxidative stress; however, plants expressing the antisense sequence of *MSRA4* were sensitive to the stress conditions (Romero et al., 2004).

In the present study, T-DNA insertion *Arabidopsis* mutant lines were screened to obtain a *salt-stress-tolerance 1* mutant (*sst1*). Further analysis indicated that the salt tolerance phenotype observed in the *sst1* dominant mutant correlates with upregulation of *AtMSRB5* (At4g04830) and *AtMSRB6* (At4g04840). We also demonstrated that overexpression of *AtMsrB5* but not *AtMsrB6* enhanced tolerance to salt stress. Arabidopsis and rice were then used as materials to generate *AtMSRB5* overexpression lines to elucidate the role of MSRB5 in salt tolerance. Of most interest, *AtMSRB5* was able to increase salt tolerance in rice without the rice homolog gene having the same function. We thus show that the potential uses of *AtMSRB5* are extensive.

## Materials and methods

### Plant materials

*Arabidopsis* T-DNA activation tagging mutants, provided by Dr. R.A. Bressan (Purdue University, West Lafayette, IN), having 4 copies of 35S enhancer (the right border within the Basta selection marker) were used. Seeds of *Arabidopsis thaliana* ecotype C24 and activation tagging mutant seeds were surface sterilized, grown in 1/2 MS medium (2.16 g L^-1^ MS salt, 1% sucrose) and incubated in a growth chamber (22°C; 16-h/8-h, light/dark; light intensity 180 μmol s^-1^m^-2^). Twelve-day-old seedlings were transferred to 1/2 Murashige & Skoog (MS) medium (Murashige and Skoog, 1962) supplemented with 150 mM NaCl. Mutant progenies with the bialaphos resistance (*bar*) gene, and single T-DNA insertion lines were selected for further studies. A sst1 mutant, was further analyzed in comparison with the wild-type C24 plant. For comparison of root length, seeds were surface sterilized and germinated in 1/2 MS for 1 week and transferred to 1/2 MS medium containing 0, 75, 100, 125, 150 or 200 mM NaCl, and kept in a vertical position for 4 days.

### TAIL-PCR

A rapid mini-preparation of *Arabidopsis* genomic DNA was conducted as described in (Chan et al., 1993). Primer design and thermal asymmetric interlaced (TAIL)-PCR followed Liu et al. (Liu et al., 1995) for the identification of the flanking region of *sst1* used for this study.

### Plasmids and gene construction

The *AtMSRB5* and *AtMSRB6* genes were isolated by RT-PCR from 10-d-old *Arabidopsis* seedlings using the specific primers covering the whole coding region (**Supplementary Table S1**). *Pfu* DNA polymerase (Promega, Madison, Wisconsin, USA) was used to amplify the DNA fragment to minimize the probability of sequence mutation. The PCR products were cloned into the pGEM-T easy vector (Promega) and sequences were confirmed by DNA sequencing analysis. The *AtMSRB5* and *AtMSRB6* cDNA fragments were excised by digestion with *Bam*HI and *Spe*I and cloned into *Bam*HI/*Spe*I site of the binary vector pCAMBIA1390/35S (Sanjaya et al., 2008). The vector was subsequently transformed into *Agrobacterium tumefaciens* strain EHA105 or GV3101 by electroporation.

### Genetic crossing, *Arabidopsis* transformation and screening

F1 mutant lines were generated by crossing the C24 and *sst1* lines. F1 progeny were screened by PCR with the B6F, B6R and LB primers as shown in **Supplemental Table S1**.

A binary vector harboring pCAMBIA1390/35S/*AtMSRB5* or *AtMsrB6* was transformed to *Agrobacterium*. The foreign gene was transferred into *Arabidopsis* by the floral dip method (Clough and Bent, 1998). Transformants were selected on MS agar plates containing 20 ppm hygromycin B and 150 ppm timentin. Transgenic plant transformed vector control was designated as 1301 and used as a negative control. The T_2_ progenies of transgenic events were collected for further analysis. To create a stacked phenotype of *AtMrsB5* and *AtMsrB6* (B5*+*B6OX), transgenic *AtMSRB5* T_2_ plants were used as parents and transformed with pCAMBIA2390/35S/*AtMsrB6* by the floral dip method, with kanamycin as selection marker. Knockout lines of *AtMSRB5* (*msrb5*) and *AtMsrB6* (*msrb6*) from the ABRC seed stock center (SALK_101496 and SALK_039712, respectively) were also used in this study. Survival rate was noted, and chlorophyll and MDA contents were tested by treatment of all genotypes with 150 mM of NaCl. The kinetic root length response to NaCl was measured by treatment with 75, 100, 125, 150 and 200 mM of NaCl.

### Subcellular localization

For subcellular localization, coding sequences of MsrB genes were subcloned into p2FGW7 (Invitrogen) to generate GFP::MsrBs fusion genes driven by the CaMV 35S promoter. Protoplasts were isolated using the Tape–Arabidopsis sandwich method and transformed using the polyethylene glycol (PEG) method (Wu et al., 2009). After incubation at room temperature for 16 h in light, protoplasts were observed with a Zeiss LSM510 META laser scanning confocal microscope.

### Generation and selection of transgenic rice

An expression vector harboring *AtMSRB5-*encoding gene under the control of the *CaMV35S* promoter was generated (**Supplementary Figure S1A**). The foreign gene was introduced into rice *via Agrobacterium*-mediated transformation (Chan et al., 1993). Following hygromycin selection, 12 putative transgenic plants, designated M1 to M12, were transferred to soil for continuous growth and seeds were harvested (T_2_ generation) for downstream studies. Genomic PCR analysis was performed to ensure the T_1_ transgenic lines were indeed harboring *AtMSRB5-*encoding gene. Five of the selected T_2_ transgenic plants (M1, M2, M4, M7 and M9) were then subjected to Southern blotting analysis (**Supplementary Figure S1B**). Both the M4 and M9 T_2_ transgenic lines were found to have a single T-DNA insertion, whereas the others harbored more than 2 copies of T-DNA insertion. Therefore, we use these three transgenic rice plants (M1, M4, M9) as the materials for the future experiments. To confirm the ectopic expression of *AtMSRB5*, Northern blot analysis was performed for M1, M4, M7 and M9 T_2_ transgenic lines. The mRNA transcripts of *AtMSRB5-Tnos* and *Hpt* were detected in the transgenic, but not the wild-type plants (**Supplementary Figure S1C**).

### RNA gel blot analysis

Total RNA was extracted using TRIZOL reagent (Invitrogen Carlsbad, CA, USA). Approximately 10 μg RNA was fractionated on 1% BTPE (10 mM PIPES, 30 mM Bis-Tris, 0.1 mM EDTA, pH 6.5) agarose gel and transferred onto nylon membranes. The filters were hybridized in 50% formamide, 1 M NaCl, 1× SSTE, 1× Denhardt’s solution, 0.2 mg ml^-1^ ssDNA at 42°C. Probes were labeled with ^32^P-dCTP by random oligonucleotide labeling. Following hybridization, membranes were washed in 2X SSC, 0.1% SDS (w/v) at 42°C, and auto-radiographed. To determine the expression pattern of AtMSRB5 and MsrB6 under salt stress, wild type was subjected to 150 mM NaCl for various times and collected for RNA gel blot analysis.

### Determination of chlorophyll content and lipid peroxidation

Chlorophyll content in leaves was determined subsequent to extraction with N,N-dimethyl formamide (Moran and Porath, 1980). Absorption of the extracts was measured at 664 nm and 647 nm. Chlorophyll content was calculated by using the following equation: total chlorophyll content = 7.04A_664nm_ + 20.27A_647nm_. The concentration of malondialdehyde (MDA), an index of lipid peroxidation, was measured in homogenates of leaves from untreated, salinity-treated, or MV-treated transgenic *AtMSRB5* rice and wild-type plants as described (Hodges et al., 1999).

### Ion content measurement

Seven-day-old seedlings grown on solid MS medium (Murashige and Skoog, 1962) were transferred to MS liquid medium. After one week, NaCl was added to reach a final concentration of NaCl of 250 mM for 0, 24 or 48 h. The seedlings were harvested, rinsed with deionized H_2_O, dried at 65°C for 2 days in an oven and weighed. Dried samples were acid-digested with Suprapur nitric acid (Merck KGaA, Darmstadt, Germany) for 16 h. Na^+^ or K^+^ content was determined by inductively coupled plasma optical emission spectrometry (ICP-OES) (Optima 5300 DV, Perkin Elmer).

### Detection of MetO content

Oxidized methionine content was determined by the CNBr cleavage method, followed by high performance liquid chromatography (HPLC) analysis. Samples of individual lines were treated with 1/2 MS (control) or 150 mM NaCl. Proteins were extracted as described previously (Ferguson and Burke, 1994). Protein samples with and without CNBr digestion underwent peptide acid hydrolysis followed by amino acid analysis by HPLC (Agilent HP1100). A ZORBAX Eclipse AAA 3.5 μM column (3.0 × 150 mm) was used, and HPLC analysis was performed following the manufacturer’s instructions.

### Real-time quantitative RT-PCR

Total RNA was prepared from various frozen plant tissues, and 3 μg RNA was used as a template to synthesize first-strand cDNA using High Capacity cDNA Reverse Transcription Kit (Applied Biosystems, Foster City, CA, USA). Quantitative real-time PCR reactions involved the use of the Power SYBR green PCR Master Mix (Applied Biosystems, Foster City, CA, USA) in an Applied Biosystems 7500 Real Time PCR System. Expression levels were normalized to *C*_T_ values obtained for *actin2* (At3g18780). The presence of a single PCR product was verified by dissociation analysis in all amplifications. All quantifications were performed in duplicate.

### Proteomic analysis

Cytosolic proteins were extracted from 10-day-old salt-treated seedlings using ice-cold protein extraction buffer [phosphate buffered saline (PBS) containing 5 mM EDTA, 1 mM PMSF, 1 mM dithiothreitol (DTT), 1X protease inhibitor cocktail (Sigma-Aldrich, St. Louis, MO, USA), 10% glycerol, and 0.01% Tween 20]. Proteins were digested with CNBr (Ogorzalek Loo et al., 1996), followed by trypsin digestion. The proteomic analysis was performed as described (Lee et al., 2014).

### Immunoblot analysis

Cytosolic proteins were extracted using protein extraction buffer. Protein samples were separated on an SDS-polyacrylamide gel, and electro transferred onto a PVDF membrane. AHAs and TIPs were recognized with H^+^ATPase antibody (Agrisera, Vännäs, Sweden) and TIP1 antibody (Agrisera, Vännäs, Sweden), respectively. Twelve percent SDS-PAGE was used for detection of AHAs and TIPs. Antibody-bound proteins were detected using a chemiluminescence system (Millipore Corporation, Billerica, MA, USA) following incubation with protein A-conjugated horseradish peroxidase (Invitrogen, Carlsbad, CA, USA).

### Ascorbate peroxidase activity

Ascorbate peroxidase activity (APX) activity was assayed according to the method described in Nakano and Asada (Nakano and Asada, 1981). Samples were determined in a reaction mixture consisting of 150 mM potassium phosphate buffer (pH 7.0), 1.5 mM ascorbate, 0.75 mM EDTA and 6 mM H_2_O_2_ by the change in absorbance at 290 nm (*E* = 2.8 mM^−1^ cm^−1^). The results were calculated in terms of micromole of ascorbate oxidized per minute.

### Statistical analysis

Data were presented as mean ± standard deviation (SD). Data were analyzed by Student’s t test or least significant difference (LSD) post hoc one-way ANOVA. The treatment means separated with use of DMRT were analyzed using SAS software (SAS Inst., Cary, NC). A P value of less than 0.05 was considered statistically significant.

## Results

### Identification of a mutant tolerant to salt stress

**Figure 1:**
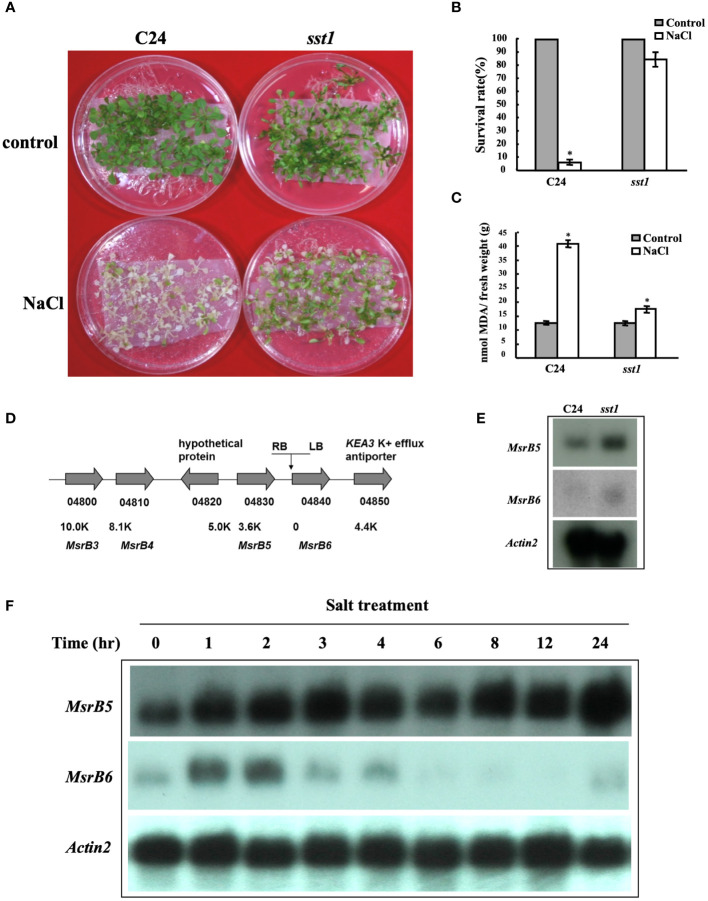
Arabidopsis salt-stress tolerance 1 (*sst1*) mutant plants are tolerant to salt stress. **(A)** Wild-type (C24) and *sst1* plants (T_2_) were grown on 1/2 MS within mesh for 11 days and transferred to 1/2 MS medium containing 150 mM NaCl for 7 days. **(B)** Their survival rate was estimated. **(C)** Malondialdehyde (MDA) content was determined after salt treatment and plants were transferred back to ½MS medium for another 3 to 4 days. **(D)** Annotation of the flanking region of the *sst1* insertion site on chromosome 4. The activation-tagging T-DNA contains 4 copies of 35S enhancer in the right border (RB) as shown. Gene annotation and distance from the insertion site are indicated. **(E)** RNA gel blot analysis of wild-type C24 and *sst1* mutant plants. Each lane contained 10 μg of total RNA. The membrane was probed with *AtMSRB5*, *AtMSRB6* and the internal control *Actin 2*. **(F)** C24 in 1/2 MS medium was transferred to 150 mM NaCl medium and sampled at different times. Total RNA was extracted and analyzed for the expression level of *AtMSRB5* and *AtMSRB6*. Data are means ± SD (n = 20) of three independent experiments. *, *P* < 0.05.

In this genetic screen, Arabidopsis T-DNA activation tagged lines were subjected to salt stress. We identified 16 *salt-stress-tolerance* (*sst*) putative mutants that were tolerant to the salt treatment conditions which were also subjected to Basta treatment (55 mg L^-1^) to confirm the presence of T-DNA insertion. A sst mutant (*sst1*) tolerant to 150 mM salt stress as compared to C24 wild type was identified and used for further in-depth analysis. As shown in **Figures 1A, B**, T2 *sst1* mutants had a similar survival rate to C24 wild-type plants under control conditions. However, under salt treatment conditions, the survival rate of T2 *sst1* was significantly higher than those of C24 wild type. Physiological analysis showed an increase in MDA content, an indicator of lipid peroxidative damage in plant tissue, in C24 wild type as shown in **Figure 1C**. These results suggested that in *sst1* the tolerance may be a result of the gain/loss-of-function of the genes associated with salt stress survival. A TAIL-PCR was performed to investigate the upstream and downstream flanking genes of the *CaMV 35S* enhancer present in the T-DNA vector and analyzed (Weigel et al., 2000). The PCR fragments were sequenced to indicate the integrated site located between *methionine sulfoxide reductase 5* (*MSRB5*) and *MSRB6* of the genome (**Figure 1D**). Based on RNA gel blot analysis, the mRNA transcripts of *AtMSRB5* were highly expressed in the *sst1*, whereas *AtMSRB6* was only slightly induced (**Figure 1E**). No differences in transcript levels were observed for the other right-border (At4g04810 (*AtMSRB4*) and At4g04800 (*AtMSRB3*)) and left-border (At4g04850) surrounding genes (**Supplementary Figure S2**). Moreover, the transcripts of *AtMSRB5* and *AtMSRB6* were induced after 150 mM NaCl treatment in wild-type C24, although *AtMSRB6* was gradually decreased after two hours post-salt treatment (**Figure 1F**). Thus, we hypothesize that *AtMSRB5* and *AtMSRB6* might be involved in the salt stress tolerance mechanism.

### Phenotype of *sst1* is linked to T-DNA insertion

**Figure 2:**
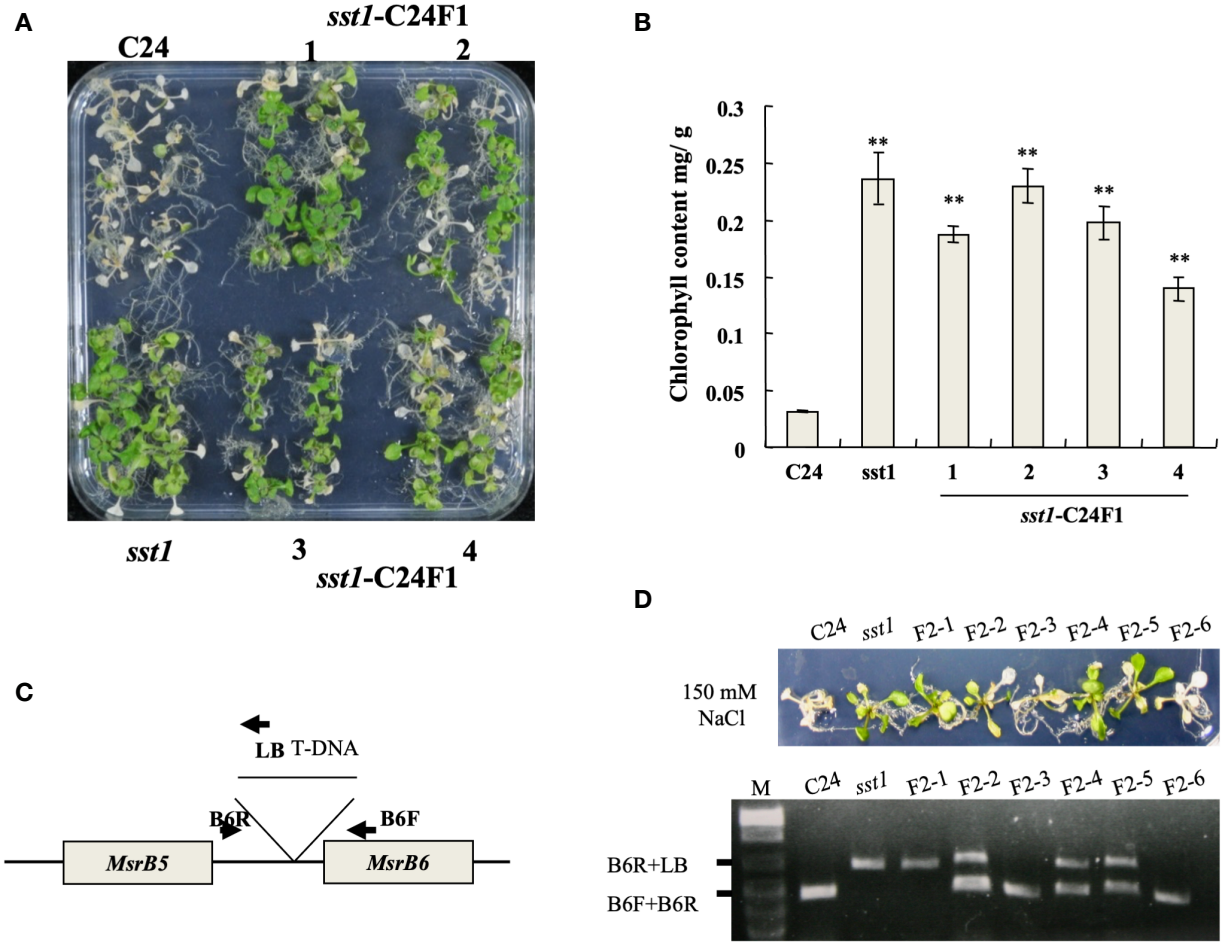
T-DNA insertion in *sst1* associated with salt-stress-tolerant phenotype. **(A)** Plants of the wild type (C24), *sst1* and their F1 progeny were treated with 150 mM NaCl; **(B)** Chlorophyll content after photographing. Data are means ± SD (n = 20) of three independent experiments. *, *P* < 0.05. **(C)** Annotation of primer and position for PCR amplification; **(D)** Salt-stress (150 mM) treatment of C24, *sst1* and six independent F2 progeny and genomic PCR with the primers is indicated in panel **(C)**. Data are means ± SD (n = 20) of three independent experiments. **, *P* <0.01.

The *sst1* mutants were crossed with C24 wild type to obtain F1 seeds. F1 progenies were germinated on plates with media supplemented with 150 mM NaCl for 1 week. Most of the plants survived (surviving/total treated plants = 138/180, 76.7%); treated *sst1* and F1 population seedlings with NaCl treatment were still green, but wild-type C24 plants turned pale with lower chlorophyll content (**Figures 2A, B**). The survival F1 were selfed to produce F2 seeds. The sst1 F2 population and wild-type C24 parents were treated with Basta/NaCl, respectively. Basta treatment resulted in three-fourths survival (surviving/total treated plants) (C24/sst1 F2 and sst1/C24 F2 survival was 208/277; 75.1% and 220/288; 76.4%, respectively); higher than the wild-type C24 (15/293; 5.1%), but lower than sst1 (269/302; 89.1%).

F2 progenies of *sst1* and C24 were treated with NaCl, and co-segregation of T-DNA was confirmed in a salt-stress-tolerant phenotype by genomic PCR (**Figures 2C, D**): surviving plants contained the homozygous or heterozygous T-DNA insertion, which indicated that the salt-stress-tolerant phenotype of sst1 is associated with the T-DNA insertion. C24, F2-3 and F2-6 plants showed no T-DNA insertion and became etiolated. Therefore, the salt-stress-tolerant phenotype of sst1 was associated with the T-DNA integration. Because both F1 and F2 progenies resemble the sst1 salt-stress-tolerant phenotype, we concluded that the T-DNA insertion of sst1 contains a dominant gain-of-function mutation in a single gene.

### Overexpressing *AtMSRB5* in Arabidopsis confers tolerance to salinity

**Figure 3:**
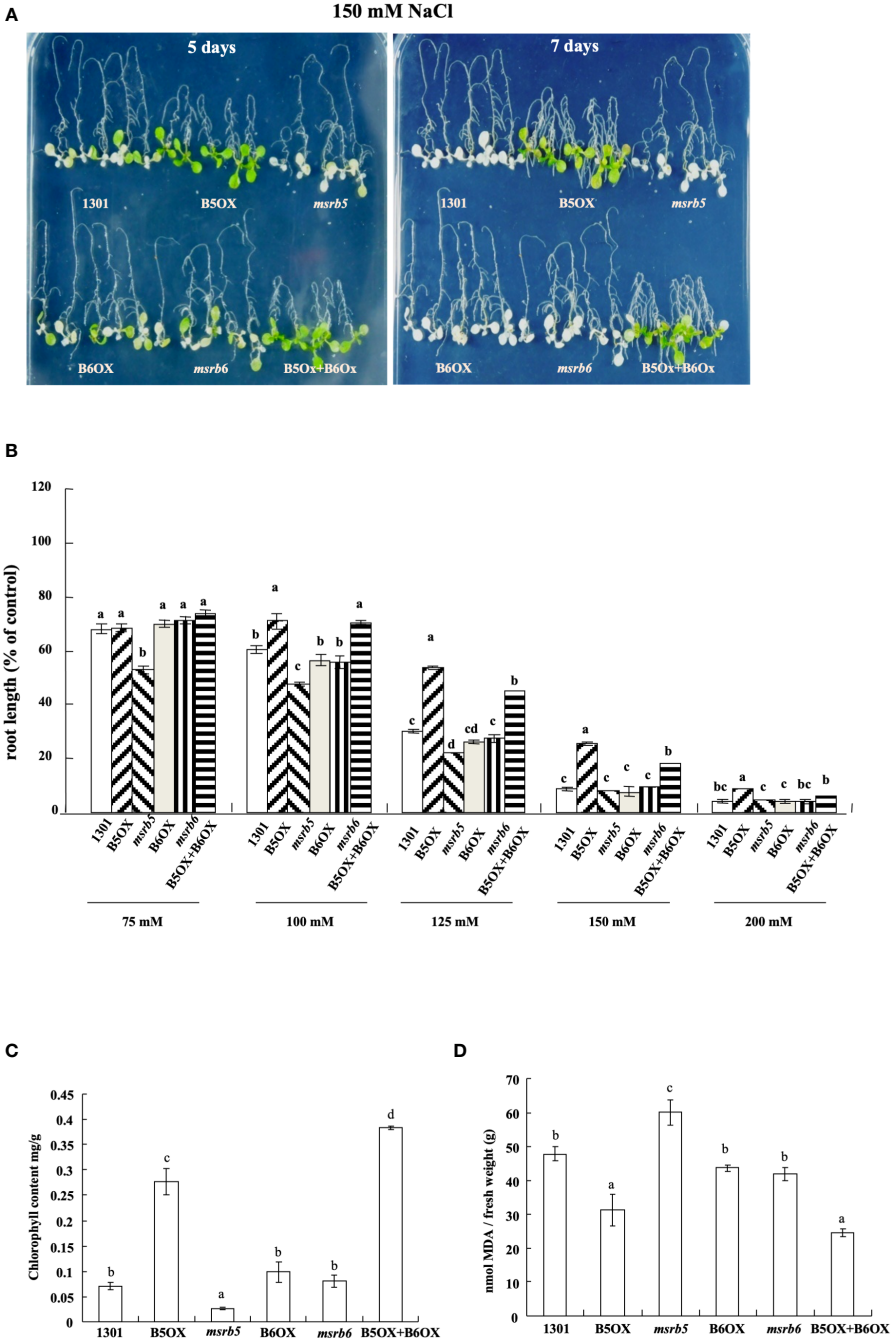
*AtMSRB5* is involved in salt stress tolerance. **(A)** Survival phenotype of plants after salt treatment. Seven-day-old *Arabidopsis* plants were treated with 150 mM NaCl for 5 or 7 days, and their survival phenotype was determined. Each construct contained at least two independent lines for stress treatment. **(B)** Comparison of relative root length growth under different concentrations of salt. Seven-day-old seedlings were subjected to different concentrations of salt treatment, and maintained vertically. The root length growth after 4 days of salt treatment was scored and compared with those grown under control conditions. **(C)** Chlorophyll and **(D)** MDA contents of individual lines after salt stress treatment for 7 days. Data are means± SD (*n* = 20) of three independent experiments and analyzed statistically using Duncan’s test. Values are means ± standard errors from 20 samples for each line in a single experiment that was repeated at least three times with similar results. Different letters above each bar indicate significant difference (LSD post hoc one-way ANOVA, *P* < 0.05).

To elucidate the roles of MSRBs in response to salinity treatment, transgenic Arabidopsis lines were generated that overexpressed *AtMSRB5* (B5OX) and *AtMSRB6* (B6OX). Arabidopsis transgenic lines co-overexpressing both *AtMSRB5* and *MSRB6* (B5OX*+*B6OX) were also generated. Homozygous T-DNA insertion mutants [*msrb5* (SALK_101496) and *msrb6* (SALK_039712)] were purchased from SALK. To examine the functional role of *AtMSRB5* and *AtMSRB6* under salt stress, 7-day-old 1301 (vector control), knockout and T_2_ transgenic plants were treated with 150 mM NaCl, and their tolerant phenotypes were examined. The transcripts of relative *MSRB5* or *MSRB6* in various plants were detected to verify the knock-out lines and transgenic plants (**Supplementary Figure S3**). We investigated the root length of plants grown under control and various degrees of salinity. As shown in **Figures 3A, B**, B5OX and B5OX*+*B6OX lines contained a relatively longer root length than other lines and were less sensitive to salt stress; moreover, *msrb5* was more sensitive than other lines under 75-, 100- and 125-mM NaCl conditions. However, the B6OX line contained a similar root length as compared to that of wild type under various NaCl conditions. B6OX line did not show enhanced tolerance phenotype. They showed the pale phenotype as similar to wild-type plants. *Arabidopsis* overexpressing *AtMSRB5* conferred resistance to salt stress, whereas the survival rate and chlorophyll content of the *msrb5* plant was considerably compromised compared to the other plants (**Figures 3C, D**). Taken together, these results indicate that *AtMSRB5* is functionally associated with salinity.

### *AtMSRB5* is involved in the modification of endogenous MetO content under saline conditions

Since MSRB functions in protein repair, next, we investigated MetO content under control and NaCl operating conditions in the wild-type and transgenic lines. Under control conditions the MetO content did not vary among the various lines; however, after 5 days of salt treatment (150 mM NaCl), lower MetO content was detected in *sst1*, B5OX and B5OX*+*B6OX plants (**Table 1**) which exhibited salt-stress-tolerant phenotypes. Furthermore, a comparison in increase in MetO content between control and B5OX line plants demonstrated that *AtMSRB5* eliminated endogenous MetO content during salinity. The *msrb5* line, on the other hand, exhibited higher MetO content. Therefore, Arabidopsis plants with overexpression of *AtMSRB*5 were more tolerant to salt stress.

**Table 1:** Determination of oxidized methionine content in overexpressing and wild-type lines under salt treatment.

	Control (%)	NaCl (%)	Fold increase*
C24	4.9 ± 0.5	40.2 ± 3.5	8.2
*sst1*	4.8 ± 0.6	24.4 ± 1.5	5.08
1301	5.1 ± 0.5	41.7 ± 4.8	8.21
B5OX	5.5 ± 0.6	20.5 ± 2.2	3.74
*msrb5*	5.6 ± 0.6	52.5 ± 3.5	9.33
B6OX	4.5 ± 0.7	37.7 ± 4.1	8.3
B6KO	5.3 ± 0.5	41.3 ± 5.1	7.74
B5OX+B6OX	5.1 ± 0.7	29.0 ± 3.2	5.7

* Fold increase was calculated by the mean values of NaCl/control. Samples of either control (1/2 MS) or salt (150 mM NaCl) treatment underwent protein extraction and oxidized methionine content was determined.

### Plants deprived of *AtMSRB5* accumulate fewer potassium ions under salt stress

**Figure 4:**
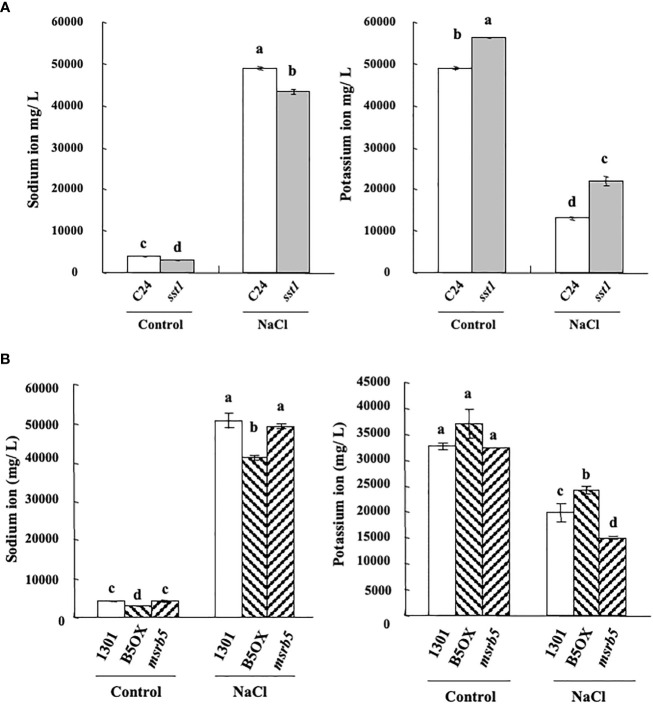
*AtMSRB5* affected potassium/sodium homeostasis under salinity. Seven-day-old seedlings were treated with 150 mM NaCl for 0 or 24 h. Samples were rinsed with distilled water, dried and collected for the detection of sodium **(A)** and potassium-ion **(B)** content. Values are means ± standard errors from 40 samples for each line in a single experiment that was repeated at least three times with similar results. Different letters above each bar indicate significant difference (LSD post hoc one-way ANOVA, *P* < 0.05).

Next, to determine the effect of *AtMSRB5* on the accumulation of sodium- or potassium- ions, endogenous K^+^/Na^+^ content was measured under control (1/2 MS medium) and saline (1/2 MS within 150 mM NaCl) conditions. As shown in **Figure 4A**, *sst1* accumulated fewer sodium ions under control and saline conditions. Similarly, *sst1* accumulated more potassium ion content than the C24 line under both control and saline conditions (**Figure 4A**). Moreover, under salt stress 1301 (vector control) and *msrb5* lines accumulated more sodium ions than B5OX. Accumulation of potassium ions was higher in the B5OX line but lower in *msrb5* under saline conditions (**Figure 4B**). Knockout of *AtMSRB5* gene in *Arabidopsis* (msr*b5)* thus decreased potassium ions to a greater extent under saline conditions. These results suggested that *AtMSRB5* has an important role in maintaining the K^+^/Na^+^ in the salt tolerance mechanism.

### Identification of putative AtMSRB5 substrates by comparative proteomic analysis using CNBr digestion

CNBr specifically hydrolyses the C terminus of Met but not MetO residues, and therefore proteins harboring MetO residues are not hydrolyzed by CNBr. Different potential substrates with *MSRB7* were identified by comparative proteomic analysis using the CNBr digestion method. For identification of the potential substrates of AtMSRB5, cytosolic protein extracts were isolated from salt treated B5OX, *msrb5* and vector control (1301) plants, followed by CNBr and trypsin digestion and analysis by LC-MS/MS. A total of 502 proteins were identified (**Supplementary Table S2**). To identify the possible interacting partners of AtMSRB5, putative targets that were present only in the proteomic data from the B5OX in B5OX/1301 and 1301 in *msrb5*/1301, or were >1.5 fold higher in B5OX than in the 1301 plants, were selected for further analysis. Analysis of the Gene Ontology (GO) annotations in TAIR (http://www.arabidopsis.org/tools/bulk/go/index.jsp) indicated that 24 of the putative targets were related to stress responses. Among them, two membrane proteins [tonoplast intrinsic protein 1;2 (TIP1;2, AT3G26520) and AHA1 (AT2G18960)] were expressed in B5OX. Thus, we proposed that these proteins might be the substrates of AtMSRB5 and be involved in salt stress and salinity-induced oxidative stress tolerance.

### AHAs but not TIPs were unstable in *msrb5* under salt stress

**Figure 5:**
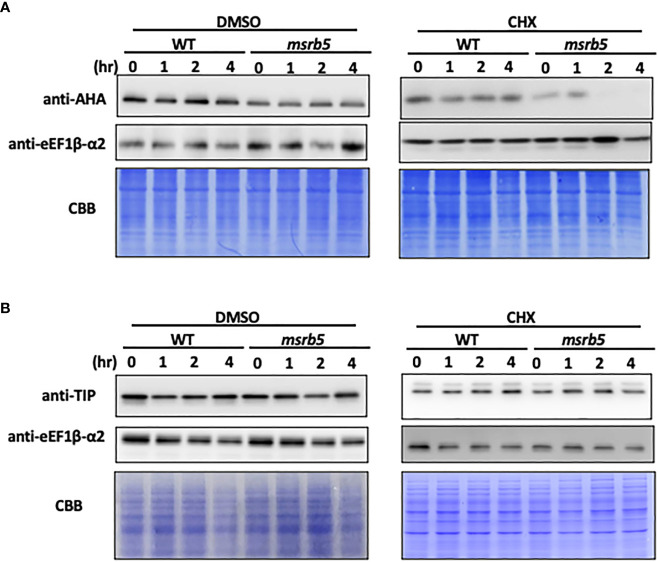
AHA but not TIP was unstable in msrb5. **(A)** C0 in 1/2 MS medium was transferred to 150 mM NaCl medium and sampled at various times. Total protein of root tissues was extracted and analyzed for the expression level of AHAs and TIPs. **(A, B)** Immunoblotting of AHA and TIP1; 2. Ten-day-old 1301 and msrb5 seedlings were pre-treated with 150 mM NaCl for 1 h followed by treatment with 0.5 mM cycloheximide for 0 to 4 h. The expression of AHAs and TIPs was detected using H+ATPase and TIP antibody, respectively. The expression of eEF1β-α2 were used as the internal control and detected using eEF1β-α2 antibody. Protein stained with CBB was used as a loading control. Twelve percent (%) SDS-PAGE was used to detect the expression of AHAs and TIPs.

Previous studies have mentioned that TIPs and AHAs are involved in the salt stress tolerance mechanism (Bose et al., 2013; Rodrigues et al., 2016). Both the TIP and AHA (TIP1;1 and TIP1;2) gene families were detected by using TIP1 antibody (Agrisera, Vännäs, Sweden) and AHA1,2,3,4,6,7,8,9,11 were detected by using H^+^ATPase antibody (Agrisera, Vännäs, Sweden). To understand the influence of AtMSRB5 on the stability of TIP and AHA proteins, ten-day-old wild-type and *msrb5* seedlings were first treated with 150 mM NaCl for 1 h followed by cycloheximide (CHX) treatment (inhibitor of *de novo* protein synthesis) for various time periods. The AHAs of the *msrb5* plants were remarkably reduced, while those in the WT plant were still accumulated after CHX treatment for 4 h (**Figure 5A**). Immunoblot analysis revealed that TIPs had no significant differences as compared to WT (**Figure 5B**). These results show that AHAs but not TIPs were not stable in the *msrb5* plant which indicates that the AtMSRB5 is involved in maintenance of AHA protein stability in plants under salt stress.

Previous studies have reported that AHA2 is localized in the plasma membrane (Bose et al., 2013; Reinhardt et al., 2016). Our subcellar localization results showed that MSRB5 and MSRB6 were localized in the cytoplasm (**Supplementary Figure S4**). To identify whether AHA interacted with AtMSRB5, bimolecular fluorescence complementation (BiFC) and yeast two hybridization assays were performed. The BiFC results indicated that AtMSRB5 did not directly interact with AHA in the plasma membrane (data not shown). Similarly, yeast two hybridization results also showed that AtMSRB5 did not interact with AHA (data not shown). Together, these results indicate that AHAs are not the direct substrates of AtMSRB5. The protein stability of AHAs through AtMSRB5 may be by other mechanism(s).

### Transgenic *AtMSRB5* rice plants exhibit tolerance to salt stress

Since *AtMSRB5* confers tolerance to salt stress in *Arabidopsis*, we evaluated whether *AtMSRB5* exhibits a similar function in rice. The rice and *Arabidopsis* genomes have been reported to contain several *MSRB* genes; three in rice (*OsMSRB1, OsMSRB3*, and *OsMSRB5*) and nine in *Arabidopsis* (*AtMSRB1-9*), respectively (Rouhier et al., 2006). To investigate whether rice *MSRB* genes are also responsive to salt, the endogenous RNA transcript levels of *OsMSRB1, OsMSRB3*, and *OsMSRB5* were semi-quantitated under salt stress conditions. As shown in **Supplementary Figure S5**, *OsMSRB1* expression levels in the aerial tissues and roots were not affected by salt stress. Expression levels of *OsMSRB3* were too low to be detected under normal and salt-treatment conditions. *OsMSRB5* expression on the other hand, was suppressed in the aerial tissues after 1 h of salt treatment; conversely, no difference was observed in the root. Since none of the *OsMSRB* genes were up-regulated upon salt treatment, the *OsMSRB* genes of rice are unlikely to be involved in salt stress tolerance.

**Figure 6:**
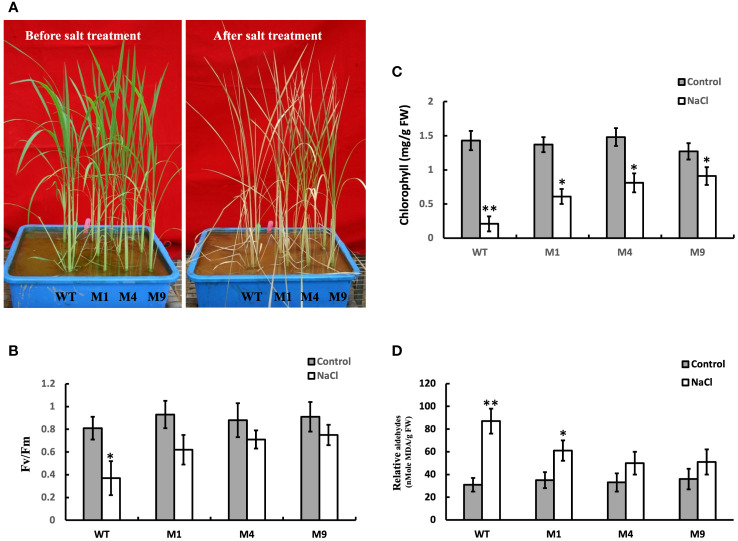
Ectopic expression of *AtMSRB5* confers rice enhanced tolerance to salt. Forty-five-day-old seedlings, comprising wild-type (WT) and transgenic *AtMSRB5* rice (M1, M4, and M9) plants, were grown on soil with or without 250 mM NaCl for 7 days. **(A)** Photographs of plants before and after salt treatment. **(B)** *F*v/*F*m ratio, **(C)** Chlorophyll content, and **(D)** MDA content, an indication of lipid peroxidation, were determined. Data are means ± SD (*n* = 6) of 3 independent experiments and analyzed statistically using student’s *t* test. *,*P* < 0.05. **,*P* < 0.01.

To investigate the tolerance levels of transgenic *AtMSRB5* rice to salt stress, wild-type and transgenic plants were subjected to salt stress (250 mM NaCl). It was observed that, after 7 days of salt treatment, leaves of the wild-type plant wilted and curled, whereas the young leaves of the transgenic *AtMSRB5* rice remained green (**Figure 6A**). Similarly, the efficiency of photosynthesis was measured using a light-induced chlorophyll fluorescence (*F*v/*F*m ratio). Under control conditions, there were no differences in *F*v*/F*m ratio and no change in chlorophyll content in either transgenic or wild-type rice (**Figures 6B, C**). When plants were subjected to salt stress, the efficiency of photosynthesis was drastically reduced in the wild-type rice, whereas the transgenic *AtMSRB5* rice was only slightly affected (**Figures 6B, C**). A measurement of the MDA content, an index of lipid peroxidation and membrane damage, suggests that the transgenic *AtMSRB5* rice was less susceptible to salt stress-induced cell damage (**Figure 6D**). These results indicated that *AtMSRB5* can functionally confer rice tolerance to salt stress by reducing the level of cell damage.

### Salt-treated transgenic *AtMSRB5* rice exhibits lower MetO content

To understand the role of AtMSRB5 in converting MetO into native Met residues, the wild-type and transgenic *AtMSRB5* rice plants were grown under salt stress and the level of intracellular MetO content was determined. Under control conditions, wild-type and transgenic *AtMSRB5* rice plants did not show any significant difference in MetO content. However, under salt treatment, the intracellular MetO content was increased by 12-fold and 7-9-fold, respectively, in the wild-type and transgenic *AtMSRB5* rice plants (**Table 2**). This difference in fold-increase observed upon salt treatment suggests that AtMSRB5 has a role in converting MetO into its native Met state, but has limited efficiency.

**Table 2:** Analysis of oxidized methionine (MetO) content in wild-type (WT) and transgenic *AtMSRB5* (M1, 4, 9) rice plants with or without 250 mM NaCl treatment.

Plants	MetO content (MetO/Met) %		Fold Salt/Control
	Control	Salt	
WT	0.4	4.8	12.0
M1	0.4	3.4	8.5
M4	0.3	2.7	9.0
M9	0.4	2.8	7.0

### Transgenic *AtMSRB5* rice accumulates fewer Na^+^ ions in leaves during salt stress

**Figure 7:**
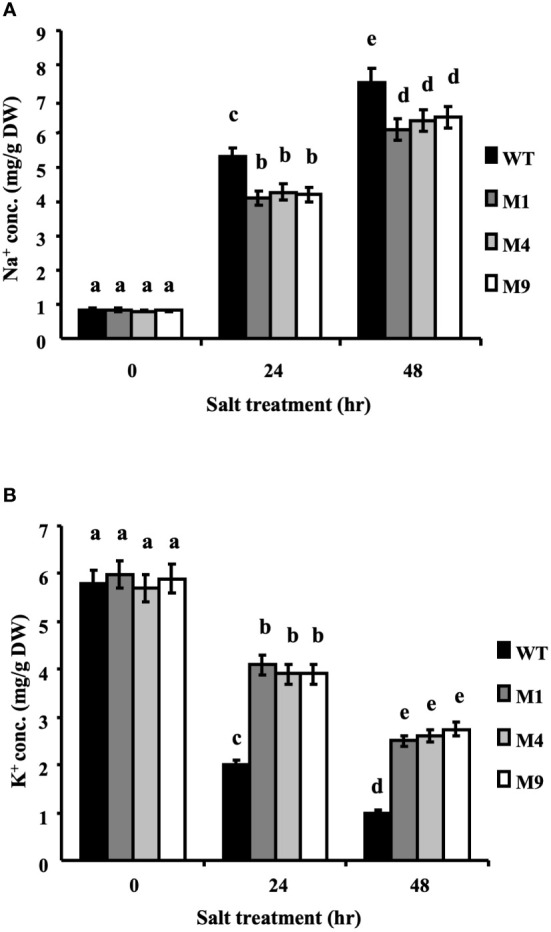
Transgenic *AtMSRB5* rice accumulated fewer sodium ions. Sixty-day-old seedlings were treated with 250 mM NaCl for 0, 24 and 48 h. Samples were rinsed with distilled water, dried, and collected. The endogenous **(A)** Na^+^ ion and **(B)** K^+^ ion contents were determined. Data are means ± SD (*n* = 3) of 3 independent experiments and analyzed statistically using Duncan’s test. Letters indicate significant differences at *P* < 0.05.

The intracellular contents of Na^+^ and K^+^ ions were determined in plants treated with 250 mM NaCl for 0, 24 or 48 h. Under control conditions, the intracellular Na^+^ and K^+^ ion contents were similar in both the wild-type and transgenic *AtMSRB5* rice plants. Upon salt treatment, the transgenic *AtMSRB5* rice exhibited significantly higher intracellular K^+^ content and lower levels of intracellular Na^+^ ion content (**Figure 7**). This suggests that the transgenic *AtMSRB5* rice maintains a better Na^+^/K^+^ ion homeostasis for tolerance under salt stress.

### Transgenic *AtMSRB5* rice maintains a higher yield than the wild type during salt stress

To determine the effect of salt stress on crop yield in genetically modified crop plants, seeds were collected from *AtMSRB5* T_2_ rice plants including the wild type to compare their yield responses (**Table 3**). Under control conditions, height of the plant, panicle number and 1000-kernel weight of the wild-type and transgenic *AtMSRB5* rice plants were similar. However, upon salt stress, the growth rate of the wild type was arrested, accompanied by the absence of inflorescences, panicles, and seed grain development. In contrast, the three transgenic lines with ectopic expression of *AtMSRB5* plants grew well and eventually transited into their reproductive stage, yielding slightly lower amount of seed grains, as compared to plants without salt stress treatment. These results suggest that *AtMSRB5* confers functional tolerance to salt stress, and does not exert any effect on rice growth and morphology.

**Table 3:** Growth characteristics of 3-month-old wild-type (WT) and transgenic *AtMSRB5* (M1, 4, 9) rice plants.

Treatment		WT	M1	M4	M9
Control	Growth days	130 ± 1	128 ± 1	134 ± 3	132 ± 2
	Height (cm)	106 ± 2	102 ± 3	108 ± 8	106 ± 4
	Panicle number	18.4 ± 0.4	18.0 ± 0.4	18.9 ± 0.4	19.0 ± 0.5
	1000-kernel weight (g)	27.4 ± 0.6	27.4 ± 0.6	26.4 ± 0.6	29.4 ± 0.5
Salinity	Growth days	60 ± 12	108 ± 4	104 ± 2	112 ± 3
	Height (cm)	76 ± 2	99 ± 5	98 ± 4	90 ± 9
	Panicle number	0 ± 0	14.5 ± 1	15.1 ± 0.4	14.3 ± 0.5
	1000-kernel weight (g)	0 ± 0	22.1 ± 1	21.0 ± 1	25.2 ± 1

Each value corresponds to the mean ± standard deviation (n = 10 plants). The stress treatment time is included in the growth period. Six-week-old transgenic AtMSRB5 and wild-type plants were treated with 250 mM NaCl for 1 month, and subsequently grown under normal conditions for another 2 weeks.

### Transgenic *AtMSRB5* rice exhibits tolerance to MV-induced oxidative stress

**Figure 8:**
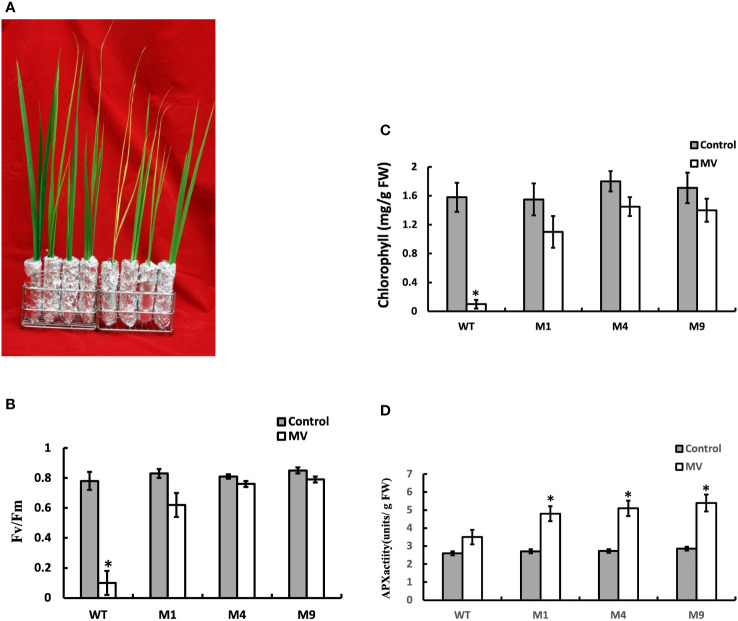
Transgenic *AtMSRB5* rice is tolerant to MV-induced oxidative stress. Sixty-day-old seedlings were subjected to 20 μM MV treatment for 7 days. **(A)** Their survival phenotype was examined. **(B)** *F*v/*F*m ratio, **(C)** Chlorophyll content, and **(D)** Ascorbate peroxidase (APX) activity were determined. Data are means ± SD (*n* = 3) of 3 independent experiments and were analyzed statistically using student’s t test. *,*P* < 0.05.

We investigated the influence of *AtMSRB5* in protecting plants against MV-induced oxidative stress. T_2_ rice plants were subjected to MV treatment, and the total chlorophyll content, *F*v*/F*m ratio, and enzymatic components of the antioxidant defense system were determined. As shown in **Figure 8A**, the leaves of the wild-type plants treated with MV were wilted, whereas those of the transgenic *AtMSRB5* rice plants remained green. In addition, a drastic decrease in the *Fv/Fm* ratio and chlorophyll content was observed in the wild-type plants, whereas a slight decrease in both parameters was observed in the transgenic *AtMSRB5* rice plants (**Figures 8B, C**) under MV treatment. Analysis of the enzymatic activity of the ascorbate peroxidase (APX; EC 1.11.1.1), an enzyme involved in the detoxification of peroxides and hydrogen peroxide (Caverzan et al., 2012), showed that the transgenic *AtMSRB5* rice subjected to salt stress was induced to express high level of the APX enzyme (**Figure 8D**). This observation suggests that *AtMSRB5* is also functionally involved in protecting rice against MV-induced oxidative stress, probably *via* the detoxification of the ROS.

## Discussion

### *AtMSRB5* plays an important role during salt stress

Abiotic stresses like drought, salt, low temperature, heavy metals, and so on are the major factors that alter crop productivity and yield (Choudhury et al., 2013). They induce the accumulation of ROS, which can damage biomolecules such as Met residues in proteins (Ezraty et al., 2005). Plants expressing AtMSRB*3* are capable of tolerating freezing *via* a reduction in the levels of endogenous MetO and ROS contents (Kwon et al., 2007). Our previous studies showed that plants overexpressing individual members of the *AtMSRB* gene family have enhanced tolerance to oxidative stress (Li et al., 2012; Li et al., 2013). These results suggested that *MSRB* genes play an important role in plant growth and environmental stress tolerance. The results of this study showed that the transcripts of *AtMSRB5* and *AtMSRB6* are salinity inducible. However, the mRNA transcripts of *AtMSRB6* were dramatically decreased during prolonged salt treatment. The mRNA levels of *AtMSRB5* were higher than those of *AtMSRB6* in roots (Rouhier et al., 2006). Constitutive expression of *AtMSRB5* or *AtMSRB6* individually and in combination indicated that *AtMSRB5*, but not *AtMSRB6*, plays an important role during salt stress. In this study, a salt tolerant *sst1* mutant was isolated and found to exhibit altered expression in *AtMSRB5* and *AtMSRB6* genes. Quantitative RT-PCR results showed that the expression of genes approximately 10 kb from the T-DNA right-border region were not affected by the enhancer. We, therefore, investigated the possible roles of these genes in salt stress tolerance. These results also implied that we may find more stress tolerance mutants from this activation tagging mutant library.

The protein-repairing function of MSRB1, MSRB2, MSRB3 and MSRB7 are reported to reduce MetO content and rescue cells from oxidative stress (from high light or MV treatment or low temperature treatment) (Kwon et al., 2007; Guo et al., 2009; Laugier et al., 2010; Oh et al., 2010; Lee et al., 2014). AtMSRB5, but not AtMSRB6, also exhibited this repair function under salinity. MetO content was higher in *msrb5* but significantly lower in *sst1*, B5OX or B5*+*B6OX plants than in other plants, which indicates that AtMSRB5 may function to repair the oxidized protein to a native form during salinity. Thus, comparative proteomic analysis using the CNBr digestion method was performed to identify the substrates of AtMSRB5. We successfully identified several putative targets which could be related to stress responses. TIPs and AHAs have been reported to be involved in the salt stress tolerance mechanism (Bose et al., 2013; Rodrigues et al., 2016). AHAs were induced by salt stress in the root (Rouhier et al., 2006). Further investigations found that AHAs but not TIPs maintained the protein stability by AtMSRB5 in the root. Moreover, B5OX and *sst1* plants accumulated fewer sodium ions but maintained a higher level of potassium ions during salinity. However, *msrb5* had no variation in sodium ion content but fewer potassium ions in comparison with 1301 vector control. Based on a study by Bose et al. (2013), high activity of the AHAs on the plasma membrane provided the proton-motive force required for maintaining better K^+^ retention (Bose et al., 2013). We suggested that B5OX plant confers enhanced tolerance to salt stress *via* maintaining the stability and activity of AHAs to increase potassium ion accumulation. However, the results of BIFC and yeast two hybridization assay (data not shown) indicated that AtMSRB5 did not directly interact with AHAs to repair the AHA protein. These results implied that AtMSRB5 may also be functionally involved in rescuing AHA proteins by other unknown mechanism(s) to maintain ionic homeostasis in transgenic *AtMSRB5* rice. ROS scavenging enzymes, GSTF2 and GSTF3, were expressed in B5OX under salt stress (**Supplemental Table S1**). GSTF2 and GSTF3 are known to maintain protein stability and activity by MSRB7 under oxidative stress (Lee et al., 2014). Thus, we propose that these proteins were stabilized by AtMSRB5 and are involved in regulating ROS homeostasis under salinity-induced oxidative stress.

Scavenging ROS has been demonstrated to play an important role in plants engineered for salt tolerance (Chinnusamy et al., 2005; Moller et al., 2007; Das, 2013). Since the MetO content of the transgenic *AtMSRB5* rice was significantly lower than the wild-type plants during salt treatment, we deduced that AtMSRB5 may also be functionally involved in maintaining the activities and stability of ROS scavenging enzymes, hence protecting/rescuing the proteins from oxidative damage. A higher level of APX activity was observed in the transgenic *AtMSRB5* rice treated with MV. APX isoenzymes play important roles in detoxifying peroxides and hydrogen peroxide, and are distributed in at least four distinct cell compartments (Jespersen et al., 1997). In *AtMARB5* transgenic rice, excess ROS might be removed by AtMARB5 *via* maintaining APX activity. However, the substrates of AtMSRB5 in rice need further investigation.

### Ectopic expression of *AtMSRB5* gene in rice enhances tolerance to salt stress

Rice is one of the most important crops in the world. With the dramatic changes in the environment, and the ever-increasing population, studies to improve rice yield are important for increasing food output. One strategy to increase rice production is to breed and select favorable phenotypes, for biotic or abiotic stresses without affecting yield. In this study, we showed that rice with ectopic expression of *AtMSRB5* is tolerant to salt- and MV-induced oxidative stresses. A comparison in the MetO content of plants treated with and without salt stress suggests that *AtMSRB5* is functionally capable of repairing the oxidized protein, but only to a certain extent. These results support the notion that overexpressing *AtMSRB5* in rice would improve tolerance to stress, without compromising yield production.

It was reported that overexpression of plastidial *Msr*, either *OsMsrA4.1* or *OsMsrB1.1*, enhanced cellular resistance to oxidative stress in yeast; in addition, *OsMsrA4.1-*overexpressing transgenic rice exhibits enhanced viability under salt treatment (Guo et al., 2009). In our study, *OsMSRB5* was found non-responsive to salt stress. Instead, the ectopic expression of cytosolic *AtMSRB5* conferred rice tolerance to salt stress (250 mM NaCl in this study), to a level that is still lower than transgenic rice overexpressing plastidial *OsMsrA4.1* (300 mM NaCl). Arabidopsis is able resist salt stress at 250 mM of NaCl (Quesada et al., 2002); however, rice is often tested at 100-200 mM of NaCl (Zhou et al., 2016; Xie et al., 2020; Jahan et al., 2021). In our experiments, we used 150 mM of NaCl for Arabidopsis while for rice with overexpression of *AtMSRB5*, we used a higher salt concentration (250 mM of NaCl) since we predicted that rice would still resist that level concentration by the overexpression of AtMSRB5. Despite having high amino acid sequence similarity (83%), our study suggests that *AtMSRB5* and *OsMSRB5* are involved in different stress tolerance mechanisms. Hazra et al. (2022) demonstrate that *OsMSRB5* is involved in maintaining seed vigor and longevity. This finding also supports our prediction. Moreover, the overexpression of homologous *GsMSRB5* is required to enhance the tolerance to high carbonate at germination and vegetative stage (Sun et al., 2016). These results imply that MSRB5 which is mainly expressed in the root may function as a protector or be involved in the repair mechanism in soil-related abiotic stress. Further studies are required to elucidate the functional role of OsMSRB5 in stress tolerance.

Overall, this study indicated that AtMSRB5 regulates ROS and Na^+^/K^+^ homeostasis *via* maintaining AHA protein stability. Likewise, overexpressing *AtMSRB5* in rice confers tolerance to salt and oxidative stresses without changing yield. The application of such gene expression strategies in crop plants may aid in crop improvement.

## Conclusion

This study demonstrated that methionine oxidation and reduction play important roles in plant salt tolerance. Therefore, we hypothesize that MsrB5 functions to improve salt tolerance by reducing oxidized methionine. We found that overexpression of *MSRB5* in plants leads to salt tolerance. MSRB5 may maintain the stability of certain proteins, which in turn leads to the development of salt tolerance in plants. Moreover, proteomic analysis revealed that AHA and TIP are affected and that AHA stability is also impaired in mutant plants lacking *MSRB5*. However, protein-protein interaction experiments confirmed that MSRBs and AHA were not directly affected. It will require further investigation to find which other protein(s) may be involved in this repair mechanism. Furthermore, an important potential application of this work is the expression of MsrB5 in rice plants that do not have a homologue of *AtMSRB5* enhanced salt tolerance. AtMSRB5 may have high potential to enhance salt tolerance in a broad range of crops of commercial importance.

## Data availability statement

The original contributions presented in the study are included in the article/**Supplementary Material**. Further inquiries can be directed to the corresponding author.

## Author contributions

DS and M-TC planned the experiments; Y-UC, J-LC, J-TL, Y-ML, FB and DS conducted the experiments and did the required measurements; Y-UC, and J-LC were responsible for preparing the first draft of the manuscript. Y-UC, M-TC, and DS revised the manuscript. All authors contributed to the article and approved the submitted version.

## Supplementary material

Supplementary Figure 1 Molecular analyses of transgenic *AtMSRB5* rice. **(A)**, Southern blot analysis of wild-type and transgenic *AtMSRB5* plants. Lanes 1 to 6: wild-type (WT), transgenic rice M1, M2, M4, M7 and M9, respectively. The radioactive-labeled *MSRB/Tnos* DNA fragment used for probing is shown. **(B)**, Northern blot analysis of wild-type and transgenic *AtMSRB5* plants. Total RNA (10 μg) was isolated from wild-type (WT; lane 1) and transgenic plants (lanes 2-5) and probed with ^32^P-labeled *AtMSRB/Tnos*, *hptII* or *OsAct1*fragment.

Supplementary Figure 2 Expression levels of genes up- and down-stream of the T-DNA insertion site. Quantitative analysis of At4g04810 (*AtMSRB4*), At4g04800 (*AtMSRB3*) and At4g04850 gene expression levels.

Supplementary Figure 3 Transcription profiles of *MSRB5* and *MSRB6* in overexpressing lines of *MSRB5* (B5OX), *MSRB6* (B6OX) and *MSRB5* plus *MSRB6* (B5OX*+*B6OX) in a Columbia ecotype were detected by qRT-PCR.

Supplementary Figure 4 Cytosolic location of AtMSRB5 and AtMSRB6 in Arabidopsis. Bar indicates 10 μm.

Supplementary Figure 5 Expression levels of *OsMSRB* genes under salt stress. Sixty-day-old rice seedlings were subjected to 250 mM NaCl treatment and samples (roots and shoots) were harvested at different time-points. RNA was extracted and the mRNA transcripts of *OsMSRB* genes in the aerial tissues **(A)**, and roots **(B)**, were quantitated by RT-PCR. Cycle numbers of RT-PCR are indicated at the right side of each panel. Os*Act1* was used for normalization. Data are means ± SD of 3 independent experiments.
