# Supplementary figures and images for: Arabidopsis AtMSRB5 functions as a salt-stress protector for both Arabidopsis and rice
Source: Front Plant Sci. 2023 Mar 22;14:1072173. doi: 10.3389/fpls.2023.1072173 (PMC10073502; doi:10.3389/fpls.2023.1072173)

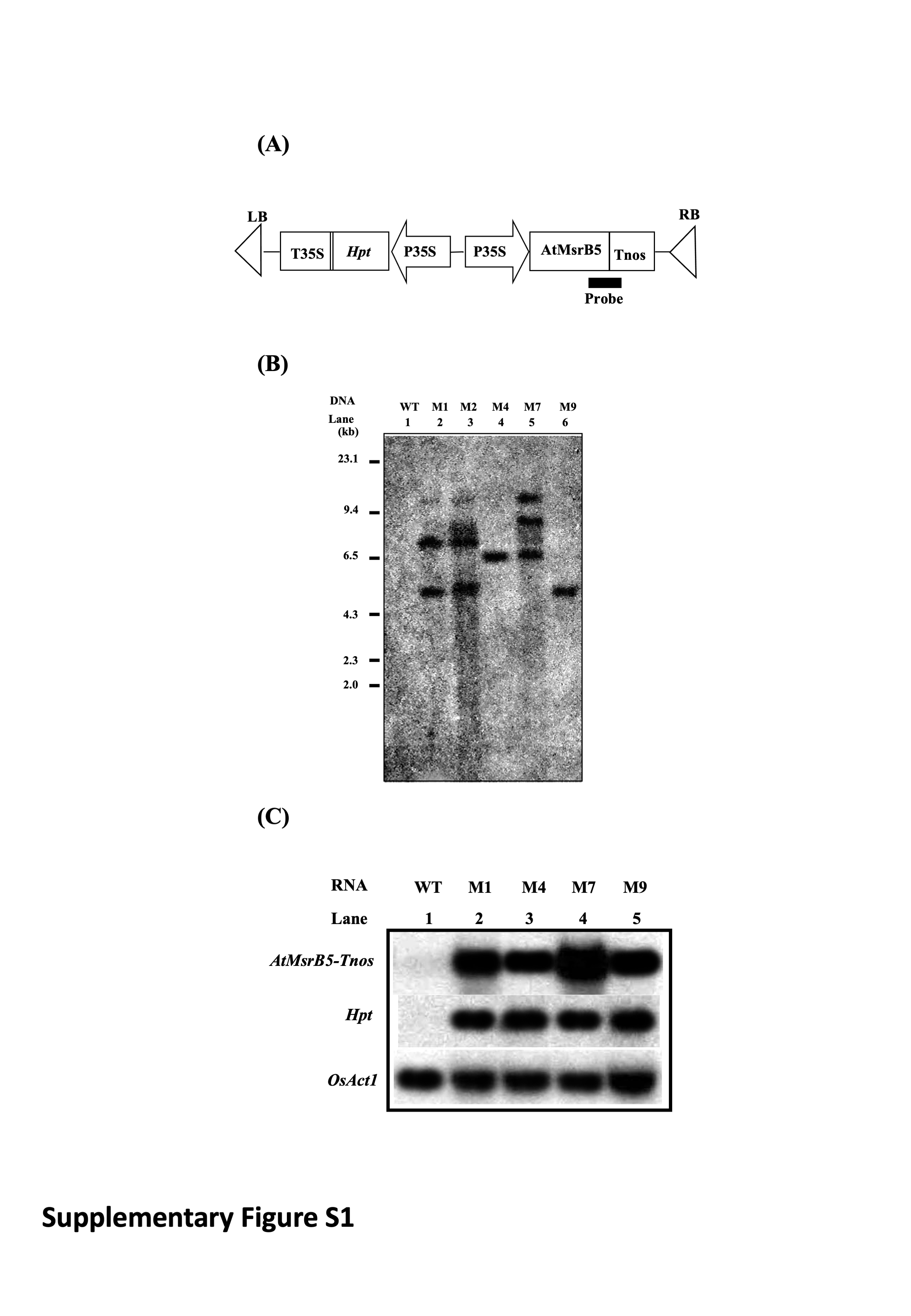

Supplement: Supplementary Figure 1 — Molecular analyses of transgenic AtMSRB5 rice. (A), Southern blot analysis of wild-type and transgenic AtMSRB5 plants. Lanes 1 to 6: wild-type (WT), transgenic rice M1, M2, M4, M7 and M9, respectively. The radioactive-labeled MSRB/Tnos DNA fragment used for probing is shown. (B), Northern blot analysis of wild-type and transgenic AtMSRB5 plants. Total RNA (10 μg) was isolated from wild-type (WT; lane 1) and transgenic plants (lanes 2-5) and probed with 32P-labeled AtMSRB/Tnos, hptII or OsAct1fragment. [file Image_1.tiff]

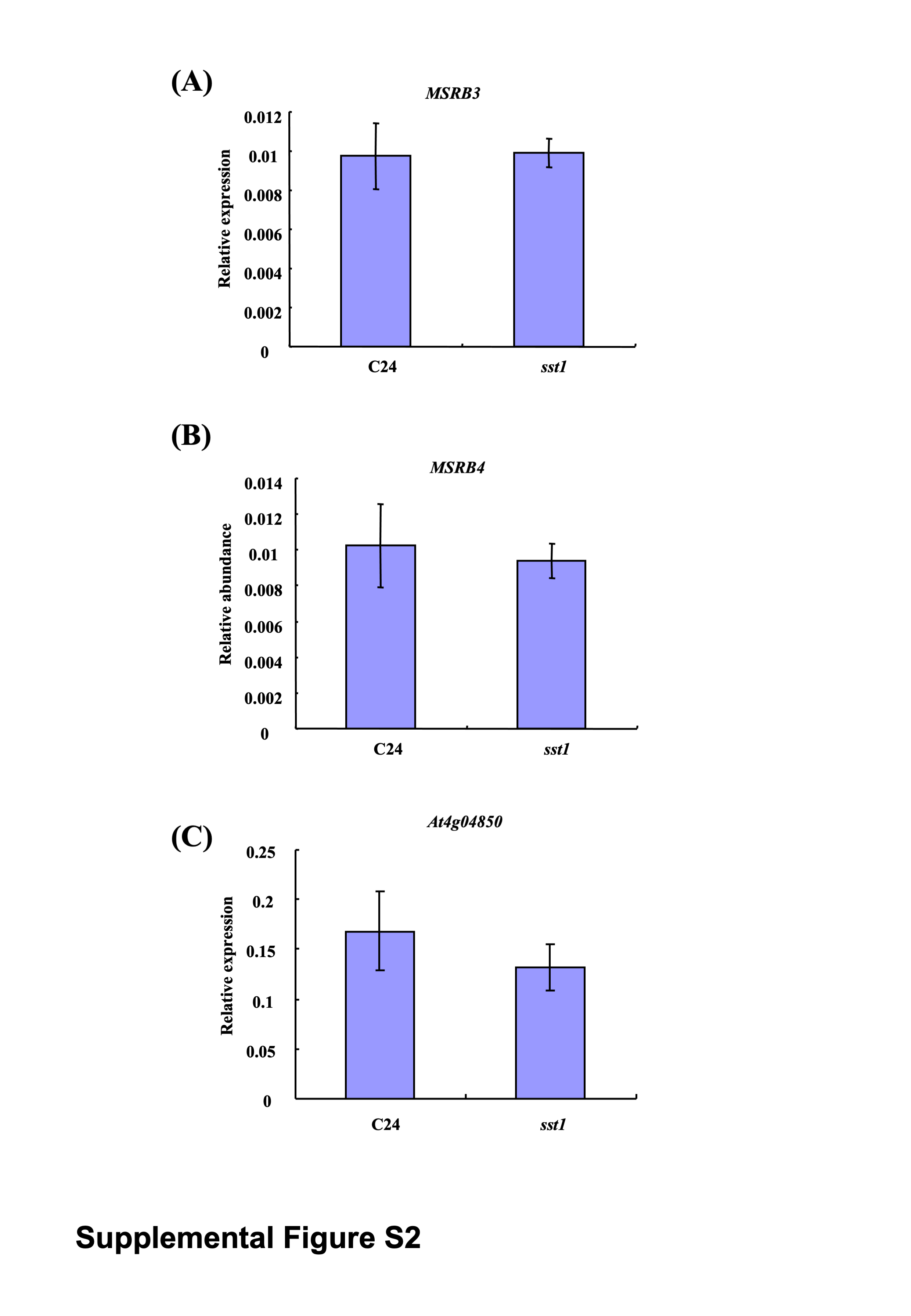

Supplement: Supplementary Figure 2 — Expression levels of genes up- and down-stream of the T-DNA insertion site. Quantitative analysis of At4g04810 (AtMSRB4), At4g04800 (AtMSRB3) and At4g04850 gene expression levels. [file Image_2.tiff]

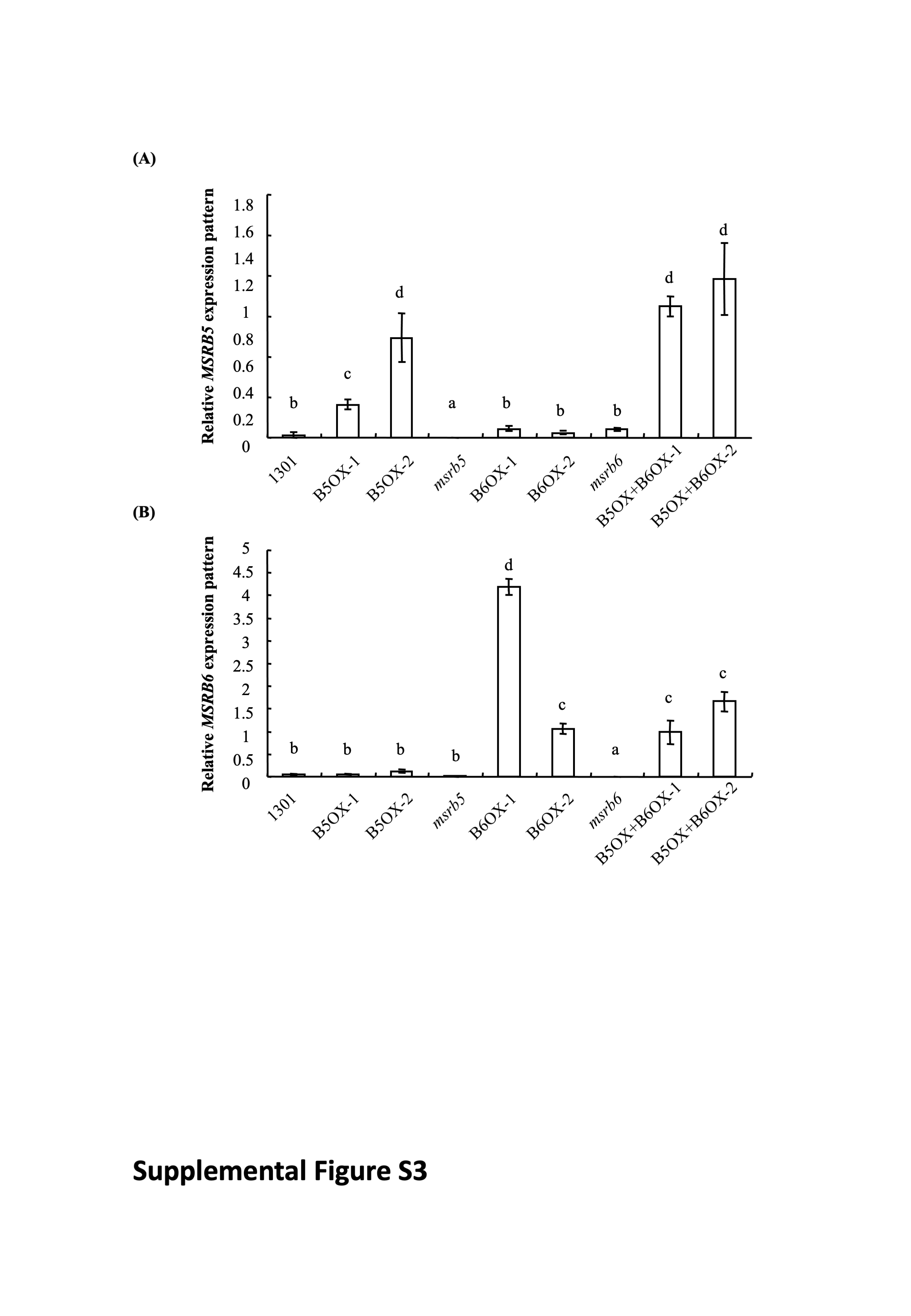

Supplement: Supplementary Figure 3 — Transcription profiles of MSRB5 and MSRB6 in overexpressing lines of MSRB5 (B5OX), MSRB6 (B6OX) and MSRB5 plus MSRB6 (B5OX+B6OX) in a Columbia ecotype were detected by qRT-PCR. [file Image_3.tiff]

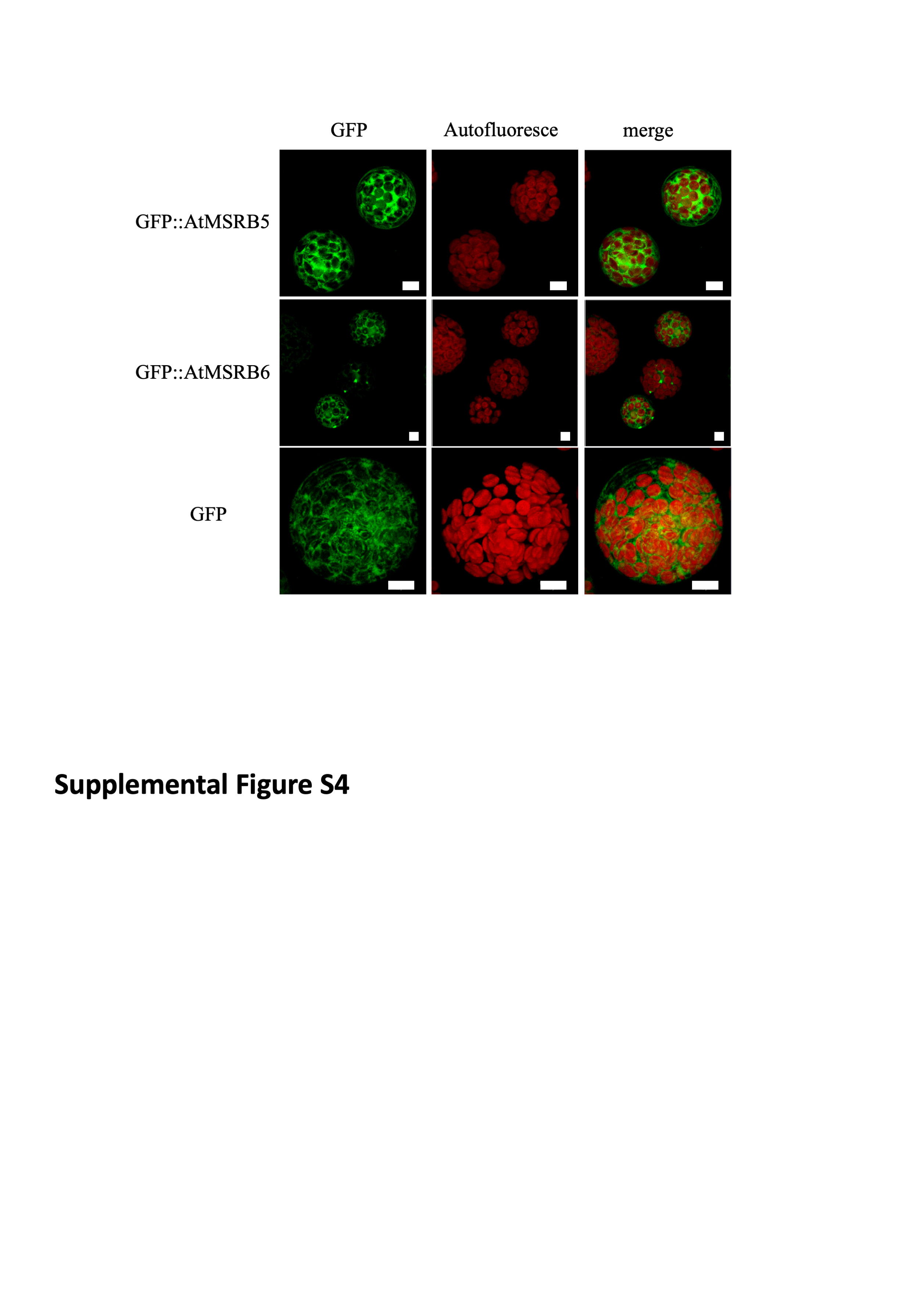

Supplement: Supplementary Figure 4 — Cytosolic location of AtMSRB5 and AtMSRB6 in Arabidopsis. Bar indicates 10 μm. [file Image_4.tiff]

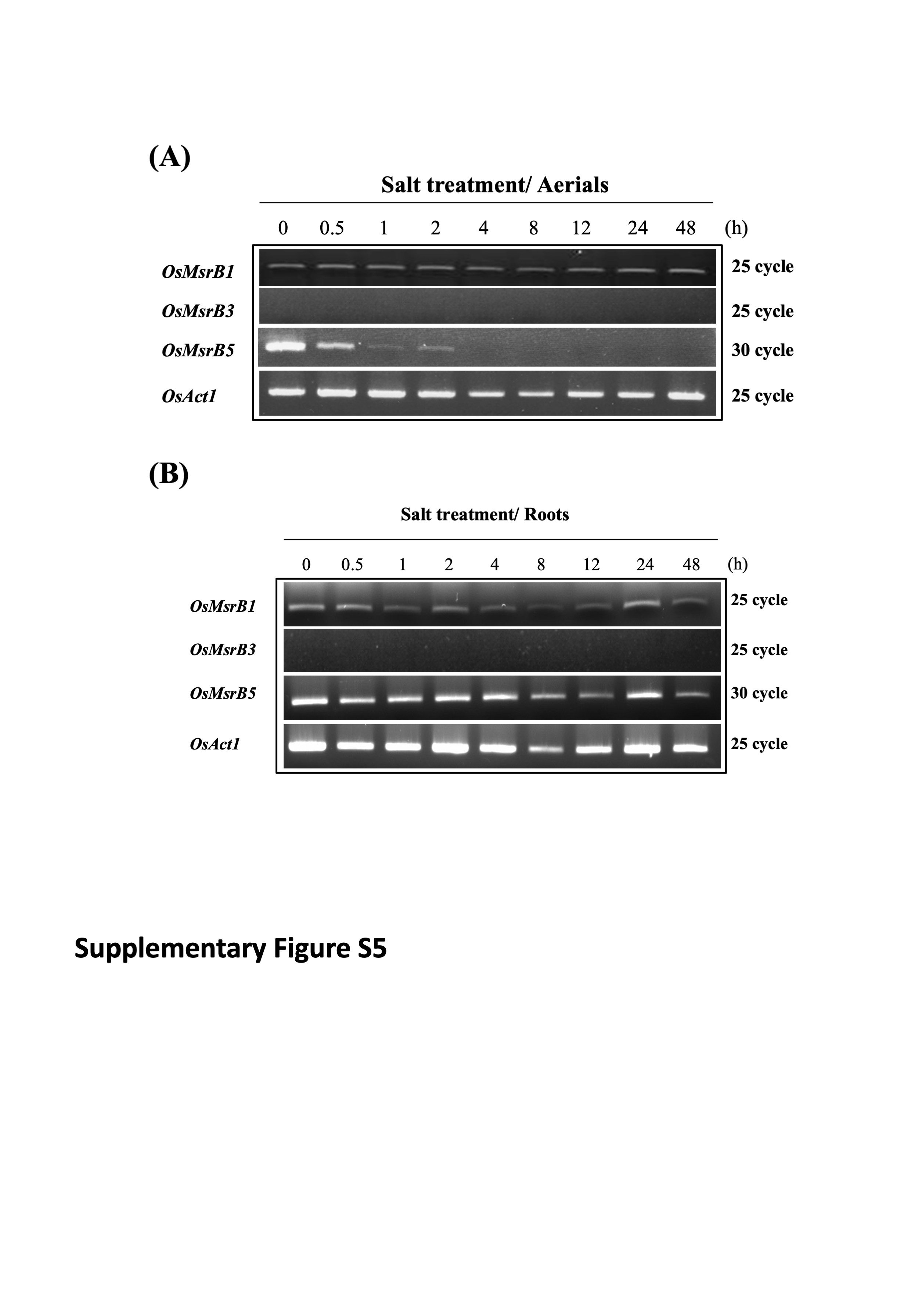

Supplement: Supplementary Figure 5 — Expression levels of OsMSRB genes under salt stress. Sixty-day-old rice seedlings were subjected to 250 mM NaCl treatment and samples (roots and shoots) were harvested at different time-points. RNA was extracted and the mRNA transcripts of OsMSRB genes in the aerial tissues (A), and roots (B), were quantitated by RT-PCR. Cycle numbers of RT-PCR are indicated at the right side of each panel. OsAct1 was used for normalization. Data are means ± SD of 3 independent experiments. [file Image_5.tiff]
