# Supplementary material for: Arabidopsis AtMSRB5 functions as a salt-stress protector for both Arabidopsis and rice
Source: Front Plant Sci. 2023 Mar 22;14:1072173. doi: 10.3389/fpls.2023.1072173 (PMC10073502; doi:10.3389/fpls.2023.1072173)
Supplement: Supplementary file 6 [file Table_1.docx]

**Supplemental Table S1.** Oligonucleotides used in PCR and/or RT-PCR.

| Gene (Loc_) | Primer ID | Nucleotide sequence |
| --- | --- | --- |
| *OsMSRB1* (Os06g27760) | OsMSRB1-F1 | CAAGCCCAAGGTAAACCGAACTA |
|  | OsMSRB1-R2 | ACAGACAGCACACAGCACCTCAGT |
| *OsMSRB3* (Os05g33510) | OsMSRB3-F1 | CCGCACTGCAACCACAACCAGT |
|  | OsMSRB3-R2 | GCGGGCTTATACTTCCATGCTC |
| *OsMSRB5* (Os03g24600) | OsMSRB5-F1 | CAACTCTCACCTACTCTGCTTCTG |
|  | OsMSRB5-R2 | CCTGGGGAATTCACCTCCAATA |
| *OsActin1* (Os03g50890) | OsAct1-F1 | CTGATGGACAGGTTATCACC |
|  | OsAct1-R2 | CAGGTAGCAATAGGTATTACAG |
| *HPT^*^* | Hpt-UP | AGCTGCGCCGATGGTTTCTACAA |
|  | Hpt-LP | ATCGCCTCGCTCCAGTCAATG |
| *AtMSRB5* | AtMSRB5-F1  AtMSRB5-R1 | GGATCCATGGCGGCTTCTCCGTTGGTGGT  ATCAGT CTGAGATGTGATAGCGGAAG |
|  | AtMSRB-F2 | CGATTGCGTAGGATGCAAGA |
|  | AtMSRB5-R3 | TATGGCACCAGGGAGTCCTT |
| *AtMSRB6* | AtMSRB6-F1 | GGATCCATGAACACTTCCCCAAAAAT |
|  | AtMSRB6-R2 | ATCAGTCTGAGATGTGATAGCGGAAG |
| *AtMSRB3*  *AtMSRB4*  At4g04850  Actin2 | AtMSRB3-F1  AtMSRB3-R1  AtMSRB4-F1  AtMSRB4-R1  At4g04850-F1  At4g04850-R1  Actin2-F1  Actin2-R1 | CTTGGCCGAACAAGATTATTTGTTC  AGTTGTTGTCAAAATGCTCGAGGTG  CTGATCCAGATGGGAGAAGAACTGAG  GTTATAGAGGGTTTGGCCGGGT  AGTTTGGCCCGTGAAAGAAAGAAAA  GCCACACAGACAGATTCTGATTGTG  ATTCAGATGCCCAGAAGTCTTGTTC  GCAAGTGCTGTGATTTCTTTGCTCA |
| pCAMBIA1390 vector | Tnos5’R | GTGGTGGTGG TGGTGGCTAG |
|  | B6R | TCATCAGAATCATGAAGCTCTCTTC |
|  | B6F | GCGTGTAAAGATGCAGCATTCT |
|  | LB | GCGTGGACCGCTTGCTGCAACT |
| AHA1 | AHA-GW-F1 | AAAAAGCAGGCTTCatgtcaggtctcgaagatatcaag |
|  | AHA1-GW-R1 | AGAAAGCTGGGTCCTACACAGTGTAGTGATGTCCTGC |
| TIP1;2 | TIP-GW-F1 | AAAAAGCAGGCTTCatgccgaccagaaacatcgc |
|  | TIP-GW-R2 | AGAAAGCTGGGTCGTAATCGGTGGTAGGCAAT |
| ^*^ HPT, hygromycin phosphotransferase.  ^**^ Tnos, Nos terminator | | |
